# Revisiting hysterical psychosis: A case report

**DOI:** 10.1192/j.eurpsy.2021.2029

**Published:** 2021-08-13

**Authors:** D. Barbosa, B. Almeida, M. Mota

**Affiliations:** 1 Psychiatry, Sao Joao Hospital and University Centre, Porto, Portugal; 2 Psychiatry, Hospital Magalhães Lemos, Porto, Portugal

**Keywords:** psychopathology, hysteria, psychosis

## Abstract

**Introduction:**

Holiender and Hirsch defined hysterical psychosis in 1964 and, while hysteria has a contemporary equivalent in somatoform/dissociation disorder, hysterical psychosis remains set adrift in the nosological understanding of psychiatric disorders.

**Objectives:**

To present a case report of a hysterical psychosis and to review this nosological construct.

**Methods:**

Clinical interview, consultation of clinical records and review of literature using the Pubmed platform.

**Results:**

The authors present a case of a 38 year-old woman, admitted in a psychiatric emergency department for bizarre behavior, restlessness, auditory (pseudo)hallucinations and emotional lability, starting 1 week after a personal development retreat. This is the second episode of this nature, the first being a 15-day hospitalization 7 years ago, with rapid stabilization, extensive examination and restitium ad integrum. The patient initiated Olanzapine and was referred to an outpatient clinic, with rapid stabilization and restitium ad integrum throughout follow-up. Given the episode and patient characteristics, a hysterical psychosis diagnosis may be accurate, taking into account the acute onset and course, the pleomorphic nature of symptoms and the presence of a disturbing life event. The authors propose reviewing the concept of hysterical psychosis regarding its clinical implications and debating its therapeutic and prognostic utility.

**Conclusions:**

Hysterical psychosis may not be a mere historic footnote and encompasses an entity with distinctive diagnostic, prognostic and therapeutic characteristics. While its etiology may not be understood, its clinical implications ensure the need for future research.

**Disclosure:**

No significant relationships.

